# Nebulized corticosteroids in the management of acute exacerbation of COPD

**DOI:** 10.4103/0970-2113.71957

**Published:** 2010

**Authors:** G. S. Gaude, S. Nadagouda

**Affiliations:** *Departments of Pulmonary Medicine, J. N. Medical College, Belgaum - 590 010, Karnataka, India*

**Keywords:** Budesonide, COPD, corticosteroids, exacerbation, nebulized steroids

## Abstract

Acute exacerbations in chronic onstructive pulmonary disease (COPD) are common and systemic steroids play an important role in the management of these cases along with the bronchodilators. Nebulized budesonide is being used in the acute attacks of bronchial asthma either in children or in adults. But the role of nebulized steroids in acute exacerbation of COPD is not much studied in the literature. In this clinical review we have evaluated the role of nebulized corticosteroids in the management of acute exacerbation of COPD (AECOPD). Through Medline, Pubmed and Embase we analyzed the various studies that has been done to study the role of nebulized corticosteroids in the management of acute exacerbation of COPD. The key words used for the search criteria were: acute exacerbation, COPD, nebulized corticosteroids, budesonide, fluticasone. Only eight studies were found which had evaluated the role of nebulized corticosteroids in acute exacerbations of COPD. All these studies had used nebulized budesonide in AECOPD in different dosages, and had been compared with both either parental or oral steroids, and standard bronchodilator therapy. All the studies had found the clinical efficacy of nebulized budesonide to be of similar extent to that of either parental or oral steroids in AECOPD. Side effects profile of nebulized budesonide was minimal and acceptable as compared to systemic steroids. Nebulized budesonide may be an alternative to parental/oral prednisolone in the treatment of acute exacerbations of COPD but further studies should be done to evaluate its long-term impact on clinical outcomes after an initial episode of COPD exacerbation.

## INTRODUCTION

Chronic obstructive pulmonary disease (COPD) is a leading cause of morbidity and mortality worldwide and results in an economic and social burden that is both substantial and increasing. COPD is the fourth leading cause of death in the United States of America (USA) and Europe.[1] Currently, COPD is a more costly disease than asthma and depending on country, 50% – 75% of the costs are for services associated with exacerbation. According to Global Initiative for chronic obstructive lung disease (GOLD), COPD is defined as a preventable and treatable disease with some significant extra pulmonary effects that may contribute to the severity in individual patients. Its pulmonary component is characterized by airflow limitation that is not fully reversible. The airflow limitation is usually progressive and associated with an abnormal inflammatory response of the lung to noxious particles or gases.[2]

Exacerbations are a common cause of morbidity and mortality in COPD patients. COPD in the USA annually accounts for 16,000,367 office visits, 500,000 hospitalizations and $18 billion in direct healthcare costs.[3] Despite aggressive medical treatment, approximately one third of patients discharged from the emergency department with acute exacerbations have recurrent symptoms within 14 days, and about 17% of patients have relapse and requires hospitalization. Identification of patients at risk for relapse improves decisions about hospital admissions and follow-up.[4]

## ACUTE EXACERBATION OF COPD

An exacerbation of COPD (AECOPD) is defined as an event in the natural course of the disease characterized by a change in the patient’s baseline dyspnea, cough, and/or sputum that is beyond normal day-to-day variations, is acute in onset, and may warrant a change in regular medication in a patient with COPD.[2] One of the earliest and most quoted definitions is that of Anthonisen,[5] which is based on an increase in symptoms of dyspnoea, sputum volume and sputum purulence with or without symptoms of upper respiratory infection and then subdivided depending on the number of symptoms present: increased sputum volume, increased sputum purulence and increased dysponea over base line. Severity of AECOPD according Anthonisen criteria[5] is: Type I: All three major criteria, Type 2: Any two of major criteria and Type 3: Any one of major criteria plus at least one of the symptom: upper respiratory tract infection in previous five days, increased wheezing, increased cough, fever without obvious source or 20% increase in respiratory rate or heart rate above baseline. This definition is based upon the infective exacerbation of the COPD patients. Patients with COPD often present with acute exacerbations of increased symptoms that frequently require a change in their usual medications. These episodes vary in severity from mild exacerbations (normally managed at home by the patient) to moderate exacerbations (requiring consultation with primary care physicians) and severe exacerbations (needing hospitalization).

## PATHOPHYSIOLOGICAL CONSEQUENCES OF AECOPD

Airflow obstruction is almost unchanged during mild exacerbations and only slightly increased during severe exacerbations. Severe exacerbations are accompanied by a significant worsening of pulmonary gas exchange (due to increased ventilation-perfusion inequality) and potentially, by respiratory muscle fatigue. Risk factors for the acute exacerbations of COPD are viral infections, bacterial infections including atypical organisms like mycoplasma and legionella, environmental pollutions including active and passive smoking, exposure to air pollution, lack of compliance with long-term oxygen therapy and bronchodilators, and failure to participate in pulmonary rehabilitation programs. Relapses in acute exacerbation of COPD are common[6] and vary between 21% and 40%; and various risk factors associated with the relapses are low pretreatment forced expiratory volume in one second, need to increased bronchodilator or corticosteroid use, previous exacerbations (more than three in the last two years), prior antibiotic treatment (mainly ampicillin), and presence of comorbid conditions (congestive heart failure, coronary artery disease, chronic renal or liver failure). Vestbo *et al*.[7] and Kanner *et al*.[8] for the first time showed that in patients with airways obstruction, exacerbations might accelerate the decline in FEV_1_. There have been several large population studies in COPD,[9–11] which shows a trivial number of exacerbations in those with mild disease (FEV_1_ >50% predicted), whereas in moderate to severe disease exacerbation rates range from 1.5 to 2.5 per year. In a prospective study of a cohort of 101 patients with moderate to severe COPD, Seemungal *et al*.[12] observed that the median number of exacerbations was 2.4 (interquartile range 1.3–3.84) exacerbations per patient per year.

## TREATMENT OF ACUTE EXACERBATION OF COPD WITH INHALED CORTICOSTEROIDS

Systemic corticosteroids play an important role in the treatment of AECOPD. If given within 24 h hour after admission for acute exacerbation, it reduces dyspnoea and improves the lung function. In a meta-analysis of Cochrane Database Systemic Review[13] it was observed that systemic corticosteroid administration (parenteral and oral) modestly reduces treatment failure rates and duration of hospitalization, and improves FEV_1_ when given to patients with AECOPD. Glucocorticoids acts at multiple points within the inflammatory cascade of AECOPD. The major effect of corticosteroids on suppression of inflammation is exerted by binding to a single class of glucocorticoid receptor, which is localized to the cytoplasm of target cells. This binding of corticosteroids to glucocorticoid receptors leads to some conformational changes in the receptor structure. Receptor–corticosteroid complex then moves into the nucleus of the target cells and binds to their DNA. This interaction changes the rate of transcription, resulting in either induction or repression of certain genes. Due to this, the production of some inflammatory cytokines, chemokines and mediators decreases while the production of some anti-inflammatory proteins and ß2-adrenoceptors increases.[14] Thus, reduction of inflammation leads to diminishing inflammatory cell infiltration, swelling and exudation within airways. Regarding the regulatory role of corticosteroids on inflammation, there are some important differences between administration of corticosteroid preparations in systemic forms and inhaled forms.[15] Data from large patient studies[9,16–18] have observed that there is an improvement in post-bronchodilator FEV_1_ and a small reduction in bronchial reactivity in stable COPD patients who were treated with oral corticosteroids. The onset of action is slow and there is little data to support a dose-response relationship. In one randomized controlled study,[18] it was observed that patients treated with oral systemic corticosteroids had fewer treatment failures, better improvement in spirometry variables and a shorter hospital stay. Furthermore, the risk of treatment failures was reduced by 10% and the average improvement in FEV_1_ was 100 ml in the first three days of treatment, compared with placebo. The study also showed that, a two week and an eight week course of systemic corticosteroids had similar clinical outcome. Therefore a shorter course of treatment, which should reduce adverse side effects, is preferred.

The exacerbation rates are significantly higher in some COPD patients, and that these patients need larger amounts of systemic corticosteroids for the control of exacerbations in a certain period of time. The major drawbacks of oral and parenteral corticosteroids are various side effects that develop during its course.[19,20] These include sleep disturbances, increased appetite, weight gain, hypothalamic-pituitary-adrenal axis (HPA axis) suppression, osteoporosis particularly in smokers, postmenopausal women and elderly, reduction in growth in children, muscle weakness, especially of the shoulder muscles and thighs, precipitation or aggravation of diabetes mellitus, redistribution of body fat, salt retention, raised blood pressure, heart failure, eye disease, particularly glaucoma and posterior sub-capsular cataracts, psychological effects including insomnia, mood changes, increased susceptibility to internal infections, especially when high doses are prescribed (e.g. tuberculosis), peptic ulcer disease, and rarely, avascular necrosis of the femoral head. The risk of development of severe adverse effects due to repeated courses of systemic corticosteroids is much higher in this subgroup, and this condition seeks clinicians to find alternative options. Inhaled corticosteroids are such an option in acute exacerbation of COPD.

Inhaled corticosteroids have a high level of topical anti-inflammatory activity and a low level of systemic activity. Mitchell *et al*.[21] compared the nebulized budesonide with oral prednisolone in the treatment of severe acute asthma and it was observed that there was no statistical difference in the clinical efficacy of 20 mg nebulized budesonide and either 30 or 160 mg oral prednisolone over 24 h. Mathew *et al*.[22] also observed that nebulized budesonide was as effective as oral steroids in improving lung function and symptoms severity in acute severe attacks of bronchial asthma in children. In another study, Devidayal *et al*.[23] studied the efficacy of nebulized budesonide compared to oral prednisolone in acute severe bronchial asthma and it was observed that oxygen saturation, respiratory rate and respiratory distress score significantly improved in the budesonide group compared to prednisolone group. The proportion of patients who were fit for discharge at the end of 2 h after the third dose of nebulization was significantly higher in the budesonide group than in the prednisolone group.

Nebulized budesonide may also be sufficiently efficacious in the management of acute exacerbation of COPD, but this has yet to be explored sufficiently in large clinical studies. Table 1 summarizes all the studies of use of nebulized budesonide in acute exacerbations of COPD. Morice *et al*.[24] studied the role of nebulized budesonide in acute exacerbation of COPD by comparing with oral prednisolone. Study group received 2 mg nebulized budesonide while the control group received 30 mg oral prednisolone as a single dose, randomized parallel-group study of 19 adults with severe acute airway obstruction due to COPD. After five days of the study, it was observed that baseline FEV_1_ increased from 1.8L to 2.1 L in the oral corticosteroid group as compared to 1.9 L to 2.0 L in the group that received nebulized budesonide, with no significant difference between two groups. All biochemical variables were similar at day one. On day five, mean urinary corticosteroid metabolites were significantly higher after nebulized budesonide: 2012 mg/24 h compared with prednisolone treatment 1079 mg/24 h (*P*< 0.05). Urinary androgen metabolites were same in both groups. The effect of treatment on serum osteocalcin was also significant 2.3 ng/ml in budesonide group compared to 0.6 ng/ml in prednisolone (*P*<0.05). Twenty four hour urinary calcium to creatinine ratio was significantly lower in budesonide treated group (0.28) compared with prednisolone treated group (0.53). This study has shown that the use of short-term parenteral corticosteroids in the treatment of severe bronchospasm causes a detrimental effect on the biochemical markers related to side effects. Nebulized budesonide treatment produced a significant improvement in these markers as compared to oral prednisolone without significant difference in FEV_1_ recovery rates.

**Table 1:** Studies showing results of utilization of inhaled corticosteroids in acute exacerbation of COPD

Authors	No of pts. studied	Treatment given	Therapeutic outcome	Side effects
Morice *et al*.[24]	19	Nebulized budesonide -2 mg bid Oral prednisolone -30 mg OD	Clinical efficacy was similar in both the groups	Urinary steroid metabolites were higher in budesonide group
Maltais *et al*.[25]	199	Nebulized budesomde -2 mg 6 hrly Oral prednisolone -30 mg 12 hrly Placebo	FEV_1_ improvement was similar to oral prednisolone Borg scale rating was similar in both groups	Higher incidence of hyperglycemia with oral prednisolone
Mirici *et al*.[26]	40	Nebulized budesomde -8 mg daily IV Preddnisolone -40 mg OD	Similar clinical efficacy as parental steroids in expiratory flow rates, PaO_2_ PaCO_2_ and SaO_2_, values	Minimal side effects
Gunen *et al*.[15,27]	159	Nebulized budesomde -1.5 mg 6 hrly Oral prednisolone -40 mg OD Standard bronchodilator therapy	Significant improvement in FVC, FEV_1_ FEF_25-75%_ and PaO_2_ in budesonide group Relapse and re-hospitalization rates were reduced by half in budesonide group	Hyperglycemia in oral prednisolone group
Wei *et al*.[28]	60	Nebulized budesomde Oral prednisolone Control group	Dyspnea score, FEV_1_ and improvement in ABG were significantly better in budesonide group	Mmimal side effects
Guozhong *et al*.[29]	40	Nebulized budesomde Control group	Better improvement in FEV_1_ and PaO_2_ values in budesonide group	Nil
Marcus *et al*.[30]	25	Budesomde suspension -0.5 mg 12 hrly MDI/DPI	Reduction in exacerbation rates Improvement in FEV_1_, better symptom control, increased confidence with budesonide	Minimal side effects
Gaude and Nemagouda[31]	125	Nebulized budesonide -2 mg bid IV Hydrocortisone -100 mg 6 hrly	Spirometry variables, SpO_2_ improvement similar in both groups	Minimal side effects
			HRQL score better improved with budesonide Duration of hospitalization shorter in budesomde group	

Maltais *et al*.[25] conducted a multicentric, randomized, placebo controlled study comparing the efficacy of nebulized budesonide, oral prednisolone and placebo in 199 patients with acute exacerbation of COPD. This was a three arm study that compared clinical efficacy of nebulized budesonide with oral prednisolone and placebo. Patients received from randomization (H(0)) to 72 h (H(72)), 2 mg of budesonide every 6 h (*n* = 71), 30 mg of oral prednisolone every 12 h (*n* = 62), or placebo (*n* = 66). All the patients received standard treatment, including nebulized beta(2)-agonists, ipratropium bromide, oral antibiotics, and supplemental oxygen. The mean change (95% confidence interval) in post-bronchodilator FEV_1_ from H(0) to H(72) was greater with active treatments than with placebo: budesonide versus placebo, 0.10 L (0.02 to 0.18 L); prednisolone versus placebo, 0.16 L (0.08 to 0.24 L). The difference in FEV_1_ between budesonide and prednisolone was not significant, -0.06 L (-0.14 to 0.02 L). It was also observed that nebulized budesonide had less systemic activity than prednisolone as indicated by a higher incidence of hyperglycemia with prednisolone group. The reduction in Borg scale ratings was of comparable magnitude in the three groups (Borg scale unit mean ± SD): budesonide 9±2.3; prednisolone, 2.6±2.3; and placebo, 1.8±2.6. The decline in PaCO_2_ was significantly greater in the two active treatment groups than in the placebo group (-1 mm Hg±4, -1 mm Hg±5, and 1 mm Hg±6, in the budesonide, prednisolone, and placebo groups, respectively (*P* < 0.05 between active treatments and placebo). Both budesonide and prednisolone improved airflow in COPD patients with acute exacerbations when compared with placebo. Thus it was concluded that high dose nebulized budesonide might be an alternative to oral prednisolone in the treatment of non-acidotic exacerbations of COPD.

In another study, Mirici *et al*.[26] compared the efficacy of nebulized budesonide with parenteral corticosteroids in the treatment of acute exacerbation of COPD. In this study, a total of 40 patients were recruited and 21 patients were administered parenteral corticosteroids treatment and 19 patients were administered nebulized budesonide treatment. Baseline characteristics of the groups were not significantly different (*P*>0.05). In each group, it was observed that increase in peak expiratory flow rate (PEFR), PaO_2_ and SaO_2_ values between the two groups were statistically significant (*P*<0.001 for all parameters). Changes in pH and PaCO_2_ values in each group were not statistically significant (*P*>0.05). It was observed that there were no significant differences between percentage changes in PEFR, PaO_2_, and SaO_2_ values during the entire period of assessment (*P*=0.75, *P*=1.00 and *P*=1.00 for PEFR, PaO_2_ and SaO_2_, respectively). This study thus demonstrated that nebulized corticosteroids had similar efficacy to systemic corticosteroids in the treatment of acute exacerbation of COPD. It was concluded that in acute attacks, a nebulized form of corticosteroids may be preferred to a systemic form because of fewer adverse effects.

The role of nebulized budesonide in the treatment of acute exacerbation of COPD was recently studied by Gunen *et al*.[27] This was a randomized, parallel group, single blind study. A total of 159 patients hospitalized with AECOPD were randomized into three groups: Group 1 received only standard bronchodilator treatment (SBDT), Group 2 received systemic corticosteroid -- 40 mg prednisolone plus SBDT and Group 3 received nebulizd budesonide - 1, 500 *µ*g sixth hourly plus SBDT. Spirometric parameters, arterial blood gases and hematological and biochemical parameters were evaluated in this study at admission, 24 h, 72 h, 7 days and 10 days. Improvement during 10^th^ day hospitalization was compared with exacerbation and re-hospitalization rates after discharge. In this study, arterial blood gas analysis and spirometric parameters [SaO_2_, PaO_2_, FEV_1_, FVC] demonstrated better improvement rates in corticosteroid groups than the only bronchodilator arm (SBDT) (*P*<0.05). More importantly, the nebulized budesonide group yielded faster return to the baseline in some of these parameters than the systemic corticosteroid group. The first statistically significant improvement in the bronchodilator only group appeared in SaO_2_ at 72 h control. However, in addition to the significant improvement rates in PaO_2_ in both corticosteroid groups at 24 h control, improvements in FEV_1_ and FVC also became statistically significant only in the nebulized budesonide group at this early control (24 h). It was also observed that mean forced expiratory flow between 25% and 75% of forced vital capacity values (FEF_25%-75%_) in Group 3 was significantly higher than the values in Groups 1 and 2 (*P*=0.03 and *P*=0.027 respectively). In addition to this, direct comparison of arterial blood gases and spirometry parameters did not reveal any difference between Groups 1 and 2, FEV_1_ was also found to be significantly higher in Group 3 than in Group 1 (*P*=0.004). Except for blood glucose level, there was no significant difference between the groups with respect to the hematological and biochemical parameters at any period. At seven-day and 10-day measurements, mean blood glucose level was found to be higher in the systemic corticosteroid group than the other groups (*P*<0.05). Early (at 10 days) and late (beyond 15 days) discharge rates did not differ between the groups (*P*>0.05). Repeat exacerbation and re-hospitalization rates within one month of discharge in the corticosteroid groups were found to be almost the half that in Group 1 (*P*>0.05). Thus it was concluded that nebulized budesonide might be an effective and well tolerated alternative to systemic corticosteroids in AECOPD.

In a study by Wei *et al*.,[28] the clinical efficacy of aerosol budesonide was evaluated in acute exacerbation of COPD. Sixty patients of acute exacerbation of COPD were randomly divided into three groups: nebulized budesonide, oral prednisolone and control group. At the completion of the study, it was observed that the dyspnoea score, FEV_1_ and improvement of arterial blood gases were significantly better in budesonide group as compared to control group. There was a statistical significant difference in clinical parameters in budesonide group as compared to control group. Budesonide group also had less systemic side effects than other groups.

In another study by Guozhong *et al*.,[29] the clinical efficacy of aerosol budesonide suspension treatment was studied in patients with acute exacerbation of COPD. Forty cases were randomly divided into two groups: control group and study group. It was observed that FEV_1_ and PaO_2_ values were higher in the nebulized budesonide group as compared to the control group. This study suggested that aerosol budesonide can improve lung functions and clinical symptoms in patients with acute exacerbation of COPD.

Marcus *et al*.[30] evaluated the role of budesonide inhalation suspension in adults with poorly controlled asthma or COPD. In this study, 25 patients with poorly controlled asthma or COPD were studied. It was observed that a transition from commonly used inhaled corticosteroid formulations administered via dry power inhaler or meter dose inhaler to nebulized budesonide inhalation suspension or initiation of inhaled corticosteroid treatment with budesonide inhalation suspension provided marked improvement in disease control for all patients and budesonide given by nebulization was well tolerated. Exacerbation rates were decreased by more than 70% in patients with asthma or COPD. Moreover, despite a long-standing history of pulmonary disease, 83% of patients with asthma and 33% with COPD demonstrated clinical improvement in FEV_1_ while receiving budesonide inhalation suspension during the one year observation period. In this study, inhaler technique was reviewed and proper inhaler use was demonstrated in the clinic setting at nearly every follow-up. A majority of patients reported an increased feeling of personal well-being, better symptom control, and increased confidence to be the main advantages of nebulizer use. Approximately 75% of patients felt their nebulizer was superior to inhalers for symptom relief.

Gaude and Nemagouda[31] conducted a parallel group longitudinal study in acute exacerbation of COPD. A total of 125 patients were included in two groups: study group received budesonide nebulization – 2 mg every 12 hourly, and control group received parental hydrocortisone -100 mg every 6 hourly. All the patients were assessed at the end of five days and at discharge with spirometry, PEFR, dyspnea grade according to MMRC, SaO_2_, and St. George Respiratory Questionnaire for health related quality of life. It was observed that nebulized budesonide had similar range of improvement in spirometry variables including PEFR and SaO_2_ as that in the control group. Patients in the nebulized budesonide group had better improvement in HRQL score as compared to control group. More number of patients in nebulized budesonide group could be discharged early as compared to control group. Thus it was concluded that nebulized budesonide was equally as efficacious as parental steroids in acute exacerbation of COPD. There were no major side effects with budesonide nebulization. There was no higher incidence of hyperglycemia in budesonide group. During the follow up period, the relapse rates for readmission for acute exacerbations were similar in both the groups. In this study, it was observed that nebulized budesonide (2 mg every sixth hourly) was equally as efficacious as parenteral / oral corticosteroids study intravenous (IV) hydrocortisone 100 mg QID /40 mg of oral prednisolone) in AECOPD. Also it was observed that nebulized budesonide reduced the duration of hospitalization and showed better improvement in HRQL as compared to parenteral /oral steroids. Overall the therapeutic outcome with nebulized budesonide in patients with AECOPD was good.

## SUMMARY

High dose nebulized corticosteroids have been tested in a limited number of studies in AECOPD. The available data suggest that nebulized budesonide might be an alternative to systemic corticosteroids in the treatment of acute exacerbation of COPD. However, as individual studies are typically underpowered and have remarkably heterogeneous methodologies, larger studies are needed to confirm these preliminary findings and determine conclusively any impact of nebulized corticosteroids in AECOPD. Nebulized budesonide may be an alternative to parental/oral prednisolone in the treatment of acute exacerbations of COPD but further studies should be done to evaluate its long-term impact on clinical outcomes after an initial episode of COPD exacerbation. Also studies are required to evaluate different types of corticosteroids with different dosages in AECOPD. Obviously, they will also make strong emphasis on the final conclusion of the place of nebulized corticosteroids in AECOPD.
